# Psychometric Properties and Rasch Validation of the Teachers’ Version of the Perception of Resources Questionnaire

**DOI:** 10.3389/fpsyg.2021.633801

**Published:** 2021-03-10

**Authors:** Ghaleb H. Alnahdi, Janka Goldan, Susanne Schwab

**Affiliations:** ^1^Special Education Department, College of Education, Prince Sattam Bin Abdulaziz University, Al-Kharj, Saudi Arabia; ^2^Faculty of Educational Science, Bielefeld University, Bielefeld, Germany; ^3^Centre for Teacher Education, University of Vienna, Vienna, Austria; ^4^Research Focus Area Optentia, North-West University, Vanderbijlpark, South Africa

**Keywords:** inclusive education, validation, Austria, Rasch analysis, resources in schools, confirmatory factor analysis

## Abstract

Research indicates that the perception of available resources is a key factor for the implementation of inclusive education. Regarding the teachers, a relatively high level of perceived personnel and physical resources is associated with more positive attitudes toward inclusive education and experiencing a higher level of self-efficacy. Thus, this study aims to examine the psychometric properties of the teacher version of the Perceptions of Resources Questionnaire (PRQ-T). Data were collected from a sample of 1,078 in-service teachers in Austria. Different statistical analyses were used, including Rasch analysis and confirmatory factor analysis. The results indicated good psychometric properties of the PRQ-T regarding internal consistency measured by person separation index (PSI) and construct validity by both confirmatory factor analysis and the Rasch analysis. Moreover, the findings supported that the PRQ-T is a two-factor scale to measure teachers’ perceptions of personnel and physical resources in inclusive education. Further studies with different samples are necessary to confirm the findings.

## Introduction

The implementation of inclusive education can be considered one of the greatest educational reforms of the century. The inclusion of various student groups, such as those with special educational needs (SEN) or migration backgrounds, into the regular school system is a major challenge for all countries (Armstrong et al., 2011; Goldan, 2019; Schwab, 2020; UNESCO, 2020). A particular issue is the provision of additional resources and the resulting—at least for a period of time—increasing costs for implementing educational reform (in the context of SEN; e.g., Peters, 2004; UNESCO, 2009; Meijer and Watkins, 2019; in the context of minority-language students; e.g., Gitschthaler et al., 2021a,b). Although personnel costs are of considerable weight, they play a key role at the teaching level, for both the students (e.g., teaching assistants and individual assistants) and the teachers (in terms of co-teaching). Recent studies reveal that the success of inclusive education depends not only on the number of available resources but also on whether these resources are perceived as sufficient (Goldan and Schwab, 2018; Schwab et al., 2020). Teachers’ attitudes toward inclusion and experiences of self-efficacy are crucial factors for success. Both constructs are affected by the perception of resources (Avramidis and Norwich, 2002).

### Measuring Perceptions of Resources

In a literature review by Avramidis and Norwich (2002), it was found that the more support a teacher receives at school, the more positive the attitude toward inclusive education in school. On average, a perceived lack of support is associated with a more negative attitude toward inclusion. Avramidis and Norwich (2002) define support services (p. 140) as resources of two kinds: physical (teaching materials, IT equipment, a restructured physical environment, etc.) and personnel (learning support assistants, special teachers, speech therapists, etc.) (Avramidis and Norwich, 2002, p. 140). In particular, the provision of additional personnel resources could significantly contribute to an increase in teachers’ acceptance of students with SEN in their classes. Moreover, extra resources led teachers to perceive the lessons as feasible, have greater satisfaction, and have a higher level of self-efficacy (Avramidis and Norwich, 2002).

Although it is evident that the provision of resources is a key factor in the success of inclusive education, it has hardly been addressed in research to date. Further, there are limited suitable constructs to comprehensively and adequately measure the perception of physical and personnel resources. Additionally, the existing constructs focus only on teachers’ perceptions and not on other stakeholders, such as students. Developing a tool for all stakeholders was necessary to investigate the extent to which the perception of resources by teachers is related to the perception of their students (Goldan and Schwab, 2018) and parents.

During the development of the scale, there were two questionnaires for teachers to assess the perception of resources. Chiner and Cardona (2013) developed an instrument with two sub-dimensions, besides “Personal support” and “Skills and resources,” which were also measured using five items. The questionnaire did not include physical and spatial resources, and there was a focus on attitudes toward inclusion, which should be investigated using the other items of their scale. It was found that attitudes are related to perceptions of available resources: the more resources a teacher perceived, the more positive their attitude toward inclusive education was.

Ahmmed (2013) also developed an instrument measuring teachers’ perceptions of perceived school support. However, the developed items were confined to the perceived support of various stakeholders in the context of teaching (e.g., principals, parents, special needs teachers, etc.); physical and spatial resources were not addressed by Ahmmed (2013).

While questionnaires for personnel resources exist, no instrument additionally considers the perception of physical and spatial resources. Thus, the Perceptions of Inclusion Questionnaire (PRQ-T) was developed (Goldan and Schwab, 2019), which measures the perception of resources from the perspective of teachers, students, and parents. It is theoretically based on a project that evaluates the costs of implementing inclusive education in Germany’s largest federal state, North Rhine-Westphalia. Local authorities were asked for several years in succession what was being acquired and what costs were incurred within the context of inclusive education (Schneider et al., 2016, 2018). The study results formed the basis for item development. In addition to physical resources, it was found that spatial resources (inclusion rooms, separées for individualized teaching) also play a decisive role in implementing inclusive education.

For the student version of the questionnaire, the pre-test revealed a two-dimensional structure, which, however, could be attributed to the direction of the item formulation. Therefore, a one-dimensional structure was adopted, which showed an acceptable internal consistency (Cronbach’s alpha = 0.74) despite the heterogeneity of the items (see Goldan and Schwab, 2018; Goldan et al., 2018). Subsequently, the items were revised and retested. In a follow-up study, the scale showed a two-dimensional structure with the sub-dimensions of “personnel resources” and “physical resources” (Goldan and Schwab, 2019; Schwab et al., 2020).

In this study, the construct validity of the teachers’ version of the PRQ-T scale (Goldan and Schwab, 2019) will be examined for the first time, using a large sample of teachers from Austria. The instrument and the accompanying instruction are reprinted at the end of the manuscript (see Appendix). It comprises 10 items, of which 6 items assess the perception of personnel resources (Items 1, 2, 3, 4, 9, and 10) and 4 items address the perception of physical and spatial resources (Items 5–8). Similar to the student version of the PRQ, a four-level response format was chosen.

To evaluate the construct validity of the PRQ-T, two main statistical analyses are applied: Rasch analysis and confirmatory factor analysis (CFA). In Rasch analysis, we fit the observed data using a unidimensional model. As “Rasch analysis is a powerful tool for evaluating construct validity” (Baghaei, 2008, p. 1,146), it will allow us to examine several measurement properties through a unified approach (Tennant and Conaghan, 2007). In addition, this study utilizes and reports on CFA of the observed data.

## Materials and Methods

### Procedure

Austria introduced German language support classes (“Deutschförderklassen”) and courses (“Deutschförderkurse”) for minority language students during the school year 2018–2019 (see e.g., Bundesministerium für Bildung, Wissenschaft und Forschung, 2019), and a study was conducted to evaluate this process. In total, primary and secondary schools from eight federal states of Austria (Vienna, Burgenland, Upper Austria, Lower Austria, Carinthia, Tyrol, Vorarlberg, and Steiermark) participated in the survey. Local school authorities of all federal states approved the ethics of the study and provided information about the schools that had introduced a German language support class and/or course; only these schools were included in the survey. In Vienna, Austria’s capital city (with the highest number of students with SEN and minority language students), the survey was conducted in paper-and-pencil format. A member of the research team^1^ contacted the school principal and delivered questionnaires for the teachers at the school. To ensure anonymity, questionnaires were collected in an envelope by team members a few weeks later. Additionally, new German language support teachers were asked to participate in the survey when they participated in teacher training, which focused on teaching minority language students. In the remaining seven federal states, the survey was conducted online using Lime Survey (Copyright^©^ 2006–2020 LimeSurvey GmbH). Only school principals were contacted and asked to forward the survey link to all school teachers. Data were collected during the winter semester 2019–2020 (September 2019–January 2020).

### Participants and Measures

The study sample comprised 1,078 teachers. The mean age of the participants was 43.40 years (*SD* = 11.86; min = 21, max = 65) and 34% were from 50 years old and above, 42% from 31 years old to 39 years old, and the rest were 30 years old or younger. The majority of the sample (around 60%) had more than 10 years of teaching experience. Around 90% of teachers were female; 78% worked in primary schools and 22% worked in secondary schools (so-called “Neue Mittelschulen,” which were educating a broad range of students and preparing for vocational training and upper secondary school). Only 2.1% of teachers worked in a so-called “Gymnasium,” which prepares students for university level, and 14.3% of the sample were working in German language tuition and support classes or courses.

#### Perceptions of Inclusion Questionnaire

The teacher version of the PRQ-T consists of 10 items, with two more items than the student version (Goldan and Schwab, 2018; Schwab et al., 2020). It was supplemented by the items “We have enough professionals to consult when I need them” and “I have enough support in class” (see Appendix for the wording of the items). The answer format in all versions has four levels, ranging from 1 = “not at all true” to 4 = “certainly true.” The psychometric structure of the scale has only been tested for the student version, in which a two-factor structure was found. Hence, it is assumed that the scale contains two factors: personnel resources with six items (e.g., “When my students have a problem, there is always someone at school to help them”) and physical and spatial resources with four items (e.g., “In the classroom we have many different materials for learning”).

#### Analyses Procedures

Two primary analyses were conducted: Rasch analysis and CFA. In the Rasch analysis, we followed the recommended steps of Tennant and Conaghan (2007). The Rasch Unidimensional Measurement Model (RUMM2030) software (Andrich et al., 2010) was used for our analysis. The partial credit model (Masters, 1982) was based on the significant likelihood ratio test (Tennant and Conaghan, 2007; Tennant et al., 2011; Vincent et al., 2015), and the thresholds were estimated for each item in this model (Andrich and Marais, 2019). Different steps were conducted in the Rasch analysis to achieve model fitness, including overall fit indices, item and person fit parameters, threshold order for all items, the unidimensionality test, the assumption of local independence in item, and differential item functioning (DIF).

CFA was used to confirm the results of the Rasch analysis. In the CFA, we aimed to identify good fit indicators within different fit indices, such as a value of >0.9 for the comparative fit index (CFI) (Hair et al., 2010) and values between0.05 and0.08 for the root mean square error of approximation (RMSEA) (Schermelleh-Engel et al., 2003). The RMSEA was lower than0.08, and the Tucker–Lewis index (TLI) was higher than 0.95 (Hu and Bentler, 1999).

## Results

First, a preliminary Rasch analysis was performed to examine whether the observed data fit the Rasch model. The results of the overall fit were supportive of the non-significant chi-square test. However, the dimensionality test result was not supportive of 15% significant tests as it was far from the recommended 5% (Smith, 2002; Tennant and Conaghan, 2007). In this test, first, a principal components analysis for the residuals was conducted (Smith, 2002; Tennant and Conaghan, 2007; Andrich and Marais, 2019). Second, based on the principal components analysis for the residuals, we got two sets of items: items that loaded positively and items that loaded negatively on the first component. Then, two ability estimates were calibrated based on these two sets of items. Next, *t*-tests were conducted to examine whether the two ability estimates were statistically significant. A 5% or less significant tests or the lower limit of 95% for the binomial proportion confidence intervals at 5% would be considered acceptable on this test (Smith, 2002; Tennant and Conaghan, 2007; Alnahdi, 2018; Alnahdi and Yada, 2020).

**FIGURE 1:**
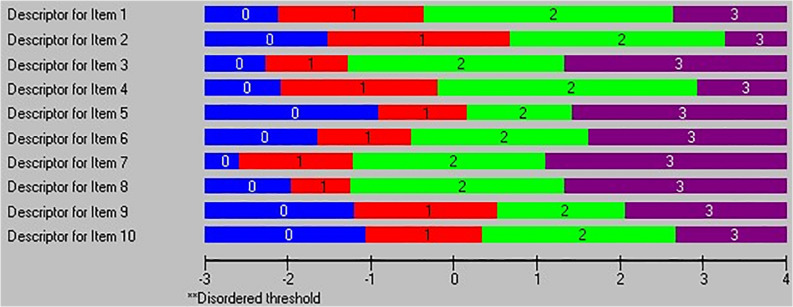
Threshold map for the overall scale.

Subsequently, we reviewed the individuals’ parameters, and 73 participants with residuals ±2.5 were removed (Tennant and Conaghan, 2007). The second run exhibited no issue with the overall fit and a non-significant chi-square. However, the dimensionality issue was not resolved, and the percentage of the significant test was still around 15%. Additionally, a threshold map was checked during the first two test runs and no issues were found; no disorder was observed for any item (see Figure 1). Next, we implemented the two subscales separately. The first subscale (personnel resources) included six items, and it indicated an improvement to fit the Rasch model in comparison with the overall scale. The chi-square test was not significant as a good indicator for the overall fit. However, the unidimensionality was not supported with the 8.6% significant *t* tests. To resolve this issue, the item residual correlation was examined and residuals from item 9 were highly correlated with different item 4 residuals. Further, a super item was created to merge items 9 and 4 and examine whether this will overcome the local dependency and improve the unidimensionality test results (Marais and Andrich, 2008a,b). Subsequently, the analysis revealed that the unidimensionality was supported with a lower limit of 95% for the binomial proportion confidence intervals of 4.6% to satisfy the recommended value of 5% or less. We then performed the Rasch analysis for the second subscale with four items. The chi-square was not significant as a good indicator for the overall fit, and the unidimensionality was also supported for this subscale (see Table 1).

**TABLE 1:** Rasch statistics at each run.

			**Item residual fit**		**Person residual fit**		**Item–trait interaction**			**Unidimensionality *t*-tests**	
	**# of items**	***N***	**Mean**	**SD**	**Mean**	**SD**	**χ^2^ (df)**	***p***	**PSI**	**% significant tests (%)**	**Lower limit of 95% CI (%)^a^**
Initial analysis	10	1,078	0.05	1.79	−0.36	1.31	120.3 (90)	0.017	0.848	15	13.4
After removing 73 misfit persons	10	1,005	0.11	1.73	−0.26	1.12	126.3 (90)	0.006	0.845	14.9	12.7
First subscale	6	1,005	−0.04	2.03	−0.35	1.05	96.56 (54)	0.000	0.827	8.6	6.9
Super item (for item 9 and item 4)	5	1,005	−0.43	3.18	−0.36	0.97	79.05 (45)	0.001	0.836	4.99	3.7
Second subscale	4	1,005	0.56	0.98	−0.36	1.04	45.62 (28)	0.019	0.672	3.1	2.1

*Bold = indicator for unidimensionality; ^*a*^ = the lower limit of 95% for the binomial proportion confidence intervals.*

**FIGURE 2:**
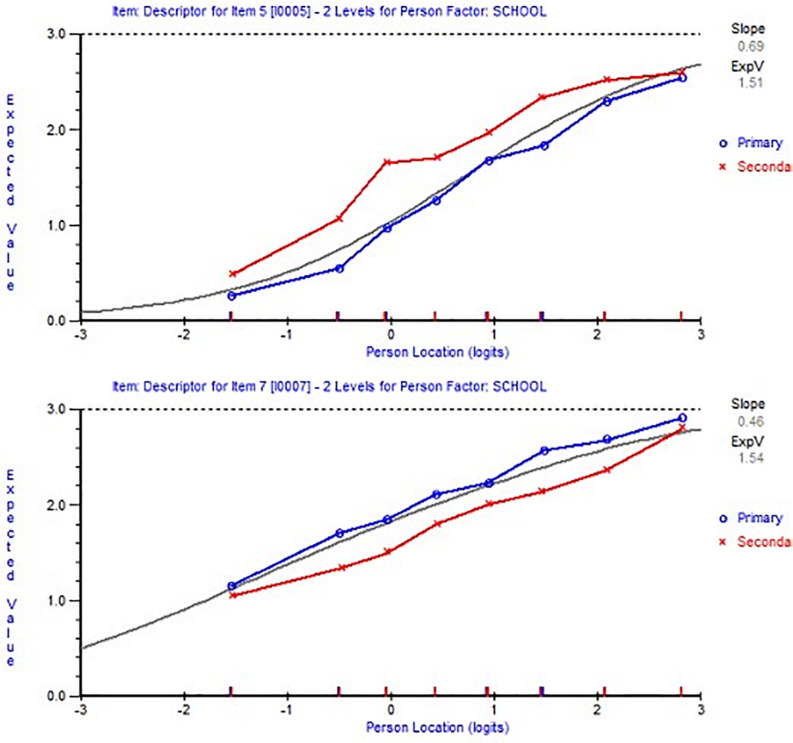
Item characteristic curves showing an example of item 7 (bottom figure) and item 5 top figure) with differential item functioning (DIF) by school.

**FIGURE 3:**
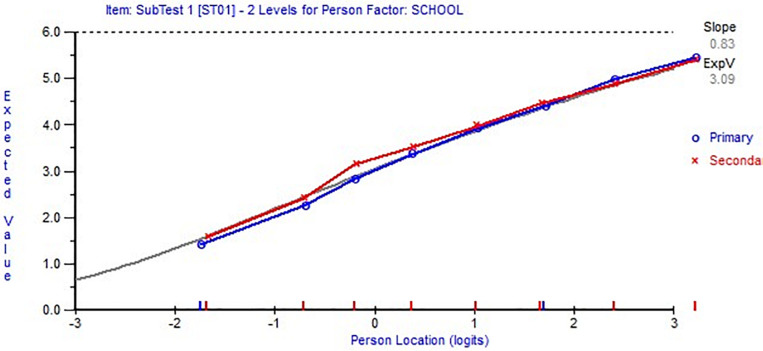
Item characteristic curves indicating no differential item functioning (DIF) by school after item 7 (bottom figure) and item 5 (top figure) grouped in one super item.

In the second subscale, two items were identified as indicators for DIF. Item 5 showed DIF to favor teachers from secondary schools, while the opposite was true for item 7 (Figure 2). To deal with this issue and to ensure that the individuals’ parameters were not affected by these items, a super item was created to group these two items and this resolved the issue (Figure 3). Each item canceled out the DIF effect of the other item, as in Figure 3, we have no differences between the two lines that represent primary and secondary teachers.

In the first subscale, two items were identified as indicators for DIF: items 1 and 10. Super items were also created to solve for DIF as in the other subscale, but this did not resolve the issue. Next, we examined the effect of the DIF on the individuals’ parameters. We performed the analysis twice, one with these two items and another without these two items to identify differences by0.6 logits or larger in persons’ locations and ensure that it does not appear in higher than 5% of the sample. The comparison of the two sets of estimations existed in 4% of the sample, which can be an indicator that the effect of DIF in items 1 and 10 was minor and no further action was needed. This was a confirmation that the scale fulfilled the requirement of the Rasch model.

**FIGURE 4:**
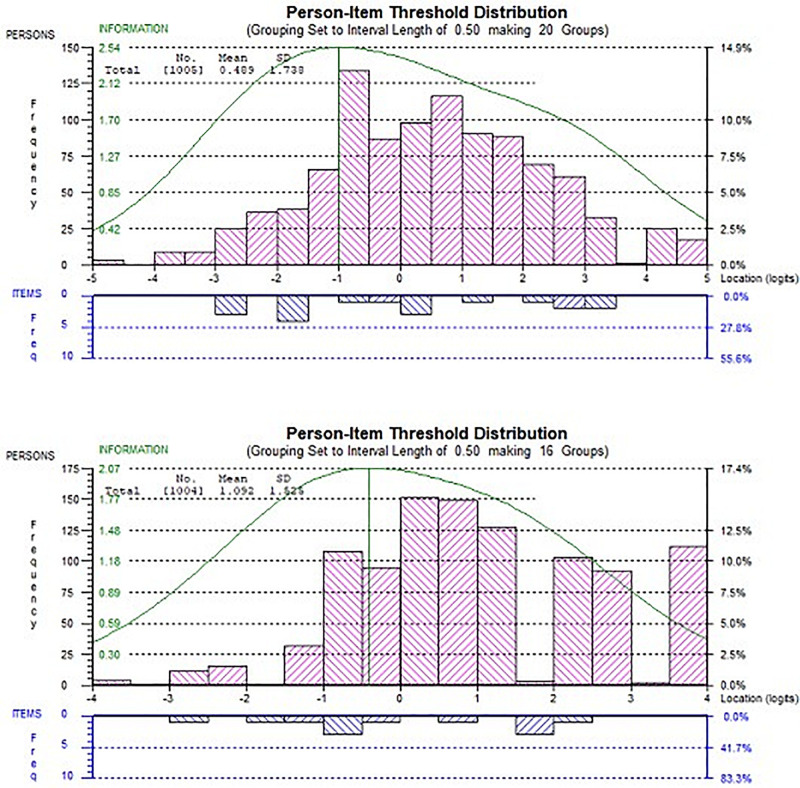
Person-item threshold plot of both subscales. The first subscale (top). In each chart, the distribution of teachers’ ability estimates (top) and item thresholds (bottom). The curve represents the information function of the scale.

The reliability of the two subscales was examined through the person separation index, and it was >0.7 as a good indicator (Tennant and Conaghan, 2007) for the first subscale and 0.67 for the second subscale, which would be acceptable when considering that it contained only four items. Figure 4 illustrates that targeting both subscales was good with low-level resources, where the range of item levels covered most levels of the participants’ abilities. In a future revision, we recommend including more challenging items, especially when targeting samples with a high perceived level of resources.

Table 2 reveals that item 2, “I have enough time for my students,” was the most difficult item to be endorsed by participants highest value in location in the first subscale (0.675). Item 3, “when my students have a problem, there is always someone at school to help them,” was the easiest item to be endorsed by participants with value in location (−1.131). While in the second subscale, item 5, “in our classrooms all students have enough space to learn,” was the most difficult item to be endorsed by participants with the highest value in location in this subscale (0.669), item 7, “In the classroom we have many different materials for learning,” was the easiest to be endorsed by participants with value in location (−0.649).

**TABLE 2:** Item fit statistics sorted based on location, from the most to the least difficult item to endorse.

**Subscale**	**Item**	**Location**	**SE**	**Fit residual**	**χ^2^**	***p*^*a*^**
1st	2	0.675	0.054	−0.886	11.905	0.219
	10	0.467	0.051	−0.781	10.615	0.303
	9	0.277	0.049	3.379	18.866	0.026
	4	−0.051	0.057	−2.029	16.177	0.063
	1	−0.237	0.056	−1.348	21.524	0.011
	3	−1.131	0.055	1.385	17.473	0.042
2nd	5	0.696	0.045	2.021	10.185	0.178
	6	0.251	0.051	−0.019	9.1	0.246
	8	−0.298	0.055	−0.062	13.967	0.052
	7	−0.649	0.056	0.329	12.375	0.089

*SE, standard error. ^*a*^Bonferroni adjusted p = 0.0083 (0.05/6).*

The item person map for both subscales in Figure 4 illustrates that the scale items covered most of the participants’ abilities (perceived level of resources). However, the second subscale revealed a greater need to include items that are more difficult to endorse, so that these could match a higher perceived level of resources.

### Confirmatory Factor Analysis

CFA was conducted to determine whether the results from CTT will support our findings in the Rasch analysis (Table 3). The first model with one factor with 10 items showed poor fit indices (CFI = 0.76, TLI = 0.69, and RMSEA = 0.16). In the second model with two factors (10 items), the fit indices improved but still did not reach the good fit indices (CFI = 0.89, TLI = 0.86, and RMSEA = 0.11). The third model was similar to the second model, but we allowed errors to correlate from items within the same subscale as it was recommended by the software to improve the fit, and it was improved to reach a good fit (e.g., CFI = 0.98, TLI = 0.97, and RMSEA = 0.05).

**TABLE 3:** The CFA Model of PRQ-T-T.

								**90% CI for RMSEA**	
**Group**	**χ^2^**	**df**	***p***	**CFI**	**TLI**	**SRMR**	**RMSEA**	**LL**	**UL**
One factor	1215.06	35	0.000	0.76	0.69	0.09	0.16	0.155	0.173
Two factors	553.99	34	0.000	0.89	0.86	0.08	0.11	0.105	0.121
Two factors*	109.58	23	0.055	0.98	0.97	0.02	0.05	0.045	0.065

CFI, comparative fit index; TLI, Tucker-Lewis index; SRMR, standardized root mean square residual; RMSEA, root mean square error of approximation; CI, confidence interval; LL, lower limit; UL, upper limit. *Errors from items within each subscale were covariate to improve the fit.

## Discussion

This study primarily aimed to examine the psychometric properties of the teachers’ version of the PRQ-T. The two-factor structure model was supported by both the Rasch analysis and CFA. In Rasch analysis, two items revealed an indicator of violation of the local independence assumption. However, that was resolved by grouping these two items as one super item, after which the unidimensionality was supported. Further, item invariance was supported for all items and only minimal DIF indicators on the item level; additionally, it was canceled out on the scale level. Therefore, we can claim that these scale items function similarly for teachers regardless of school level. In sum, this study supports that the PRQ-T is a two-factor scale to measure teachers’ perception of resources for inclusive education.

From the research perspective, it can be assumed that comprehensive and inclusive education is more cost-effective than systems with predominantly exclusionary school models (e.g., special needs or special schools) (e.g., Peters, 2004; UNESCO, 2009). This is particularly true when considering the long-term costs to society (e.g., in terms of unemployment rates or follow-up costs in the health care system) (e.g., Schwab, 2020). However, regular schools must be equipped with additional (monetary) resources so that additional materials, sufficient rooms, and above all, personnel are available for special individual needs of young people with special support needs (e.g., special educational or language support) to receive appropriate assistance. However, it is crucial that not only additional resources are made available for regular schools, but that these resources are also used cost-effectively and flexibly. Regarding the aspect of flexibility, it is difficult to introduce general regulations (e.g., the direct linking of a certain amount of money for a child diagnosed with SEN). Therefore, the following question arises: When are resources perceived as sufficient by key players in the field (e.g., teachers, students, and their parents) and what factors are associated with this sufficiency?

Recently, it has become increasingly evident, especially via qualitative research, that lacking resources are a barrier to high-quality (inclusive) education (e.g., Armstrong et al., 2011; Schwab, 2018). However, evidence revealed that lacking resources might be perceived as not only objective aspects (e.g., in terms of how much money a state spends on education) but also subjective factors. Different teachers might perceive identical objective resources as sufficient or insufficient. Therefore, it is important to investigate teachers’ perceptions of resources and investigate the factors that are related to this. To close this research gap, it is necessary to have a reliable and valid scale that assesses teachers’ subjective perceptions of available resources.

In sum, the main contribution of this study is that it is the first to investigate the psychometric qualities of the teacher version of the PRQ-T scale with a sufficient sample. While the previous studies of Goldan and Schwab (2018) and Goldan et al. (2021) mainly focused on students’ perceptions of resources and the psychometric qualities of the students’ version of the PRQ-T (and only included a very limited number of teachers), this is the first study to demonstrate that the teacher version has acceptable psychometric properties to measure teachers perception of resources. In addition, it is important for future studies to have different samples from different populations to confirm the findings of this study and examine whether the teacher version of the PRQ-T scale will have good psychometric properties across cultures.

### Implications and Future Research

By having reliable and valid measures to regard teachers’ perceptions of resources, it will be possible in the future for researchers to examine how strongly teachers’ perceptions of resources are linked with their teaching quality using this instrument. Further, an analysis of how their perception of resources can be fostered and how many resources are needed based on teachers’ perceptions can be performed. Schools can use the instrument to identify teachers’ individual needs (which might greatly differ across class levels) and local school boards could use it to identify which schools might have more needs compared to others. Owing to the implementation of inclusive education, the requirements and demands for schools have undoubtedly increased. It is also undisputed that sufficient additional resources are indispensable to ensure high-quality inclusive schooling. Therefore, the findings presented here are not intended to imply that all that is needed to successfully implement inclusive education is to change teachers’ perceptions of resources. The task of future studies is to determine the extent to which additional resources influence perception. The decisive factor will not primarily be how many resources are available, but whether they are used efficiently. If schools can achieve this with fewer resources, quantitatively speaking, and there is a perception among teachers that sufficient resources are available for inclusive education, then this represents a significant gain. Conversely, for a school that is better equipped but whose teachers still perceive resources as very few for school inclusion (because they are used inefficiently), simply continuing to add (personnel or material) resources is unlikely to achieve much.

The perception of existing resources has been shown to influence teachers’ attitudes toward inclusion and their self-efficacy beliefs (Chiner and Cardona, 2013; Schwab, 2018)—two factors that have been used for years to explain the success of inclusive education. The PRQ-T developed in this study provides a starting point for exploring the importance and role of resource provision at the teacher and student levels (Goldan and Schwab, 2018) in inclusive education in the future. In terms of policy making and school management, the PRQ-T provides an instrument that allows decision makers (e.g., education ministries and principals) to evaluate whether teachers consider the provision of resources as sufficient. This can help to identify and prevent the imminent risk of teachers being overworked or even burnt out, at an early stage.

## Data Availability Statement

The datasets generated for this study are available on request to the corresponding authors.

## Ethics Statement

The studies involving human participants were reviewed and approved by the local school authorities of all federal states approved the ethics of the study and provided information about the schools that had introduced a German language support class and/or course. The patients/participants provided their written informed consent to participate in this study.

## Author Contributions

All authors contributed to the article and approved the submitted version. GA did the calculation and analysis part and wrote the results section. JG and SS wrote the introduction part. SS managed the data collection. SS and GA wrote the discussion section. The data collection took place in Austria.

## Conflict of Interest

The authors declare that the research was conducted in the absence of any commercial or financial relationships that could be construed as a potential conflict of interest.

## Footnotes

1 The study was carried out with support of several university students supervised by Susanne Schwab. Data collection and data entry were supported by Raffaela Berenjchi, Katharina Brammen, Kerstin Buschnegg, Asiye Coskun, Christina Debu, Thomas Gril, Rosalie Pirker, Sandra Waltl, Tanja Ganotz and Julia Kast.

## Appendix

### Instruction for Teachers

In the following, we would like to know how you perceive the provision of resources at your school. Please indicate to what extent you agree with the statements.

**Table:**

		**Not at all true**	**Somewhat not true**	**Somewhat true**	**Certainly true**
1.	In class, all students get the help they need.	□	□	□	□
2.	I have enough time for my students.	□	□	□	□
3.	When my students have a problem, there is always someone at school to help them.	□	□	□	□
4.	In class, all students get the support they need to learn.	□	□	□	□
5.	In our classrooms, all students have enough space to learn.	□	□	□	□
6.	We have all the materials we need for our lessons.	□	□	□	□
7.	In the classroom, we have many different materials for learning.	□	□	□	□
8.	Our classrooms are designed so that the students feel comfortable.	□	□	□	□
9.	We have enough professionals to consult when I need them.	□	□	□	□
10.	I have enough support in class.	□	□	□	□
